# Tryptophan Oxidation in the UQCRC1 Subunit of Mitochondrial Complex III (Ubiquinol-Cytochrome C Reductase) in a Mouse Model of Myodegeneration Causes Large Structural Changes in the Complex: A Molecular Dynamics Simulation Study

**DOI:** 10.1038/s41598-019-47018-6

**Published:** 2019-07-23

**Authors:** Sruthi Unni, S. Thiyagarajan, M. M. Srinivas Bharath, B. Padmanabhan

**Affiliations:** 1 Department of Biophysics, National Institute of Mental Health and Neurosciences (NIMHANS), Hosur Road, Bangalore, 560029 Karnataka India; 2 Institute of Bioinformatics and Applied Biotechnology (IBAB), Biotech Park, Electronic City Phase I, Electronic City, Bangalore, 560100 Karnataka India; 3 Department of Clinical Psychopharmacology and Neurotoxicology, NIMHANS, Hosur Road, Bangalore, 560029 Karnataka India; 4 Neurotoxicology Laboratory at the Neurobiology Research Center, NIMHANS, Hosur Road, Bangalore, 560029 Karnataka India

**Keywords:** Protein function predictions, Protein structure predictions

## Abstract

Muscle diseases display mitochondrial dysfunction and oxidative damage. Our previous study in a cardiotoxin model of myodegeneration correlated muscle damage with mitochondrial dysfunction, which in turn entailed altered mitochondrial proteome and oxidative damage of mitochondrial proteins. Proteomic identification of oxidized proteins in muscle biopsies from muscular dystrophy patients and cardiotoxin model revealed specific mitochondrial proteins to be targeted for oxidation. These included respiratory complexes which displayed oxidative modification of Trp residues in different subunits. Among these, Ubiquinol-Cytochrome C Reductase Core protein 1 (UQCRC1), a subunit of Ubiquinol-Cytochrome C Reductase Complex or Cytochrome b-c1 Complex or Respiratory Complex III displayed oxidation of Trp395, which could be correlated with the lowered activity of Complex III. We hypothesized that Trp395 oxidation might contribute to altered local conformation and overall structure of Complex III, thereby potentially leading to altered protein activity. To address this, we performed molecular dynamics simulation of Complex III (oxidized at Trp395 of UQCRC1 vs. non-oxidized control). Molecular dynamic simulation analyses revealed local structural changes in the Trp395 site. Intriguingly, oxidized Trp395 contributed to decreased plasticity of Complex III due to significant cross-talk among the subunits in the matrix-facing region and subunits in the intermembrane space, thereby leading to impaired electron flow from cytochrome C.

## Introduction

The skeletal muscle activity is critically dependent on the metabolic status, redox balance, and mitochondrial function^1^. Studies in experimental models and human muscle biopsies have indicated that muscle diseases display mitochondrial dysfunction and oxidative damage. Our previous study^2^ in a cardiotoxin (CTX) model of myodegeneration correlated with muscle damage and cell death with morphological and biochemical alterations in the muscle mitochondria along with oxidative damage. Mitochondrial proteomics in the CTX model demonstrated down-regulation of critical proteins contributing to energy metabolism, including respiratory complexes and Krebs cycle among others. Muscle biopsy from human muscle pathologies: Dysferlinopathy (Dysfy) [representing Muscular Dystrophy (MD)], Distal Myopathy with Rimmed Vacuoles (DMRV) and Polymyositis (representing inflammatory myopathies), and Lipid Storage Disease (LSDs) (representing metabolic disorders) with varied pathology, disease severity and clinical outcome, revealed morphological and biochemical changes in the mitochondria and differential expression of mitochondrial proteins, as revealed by proteomics^3,4^. Mitochondrial dysfunction in muscle pathologies is associated with oxidative stress and oxidative post-translational modification of proteins. Our previous study^5^ demonstrated that protein oxidation directly correlated with the severity of muscle pathology with Duchenne muscular dystrophy (DMD) displaying highest carbonylation of cellular proteins. Protein oxidation was also observed in muscle biopsies from DMRV, PM, and Dysfy patients^3^. Proteomic identification of oxidized proteins in DMD human muscle revealed specific mitochondrial proteins targeted for protein oxidation^2^ confirming that mitochondrial dysfunction and chronic oxidative damage could contribute to muscle diseases.

Post-translational oxidative modification of cellular proteins is linked with aging and disease^6^. Several amino acids are vulnerable to oxidation with Cys and Trp being the most frequently oxidized amino acids. Trp oxidation among mitochondrial proteins has been documented based on the mining of mass spectrometry (MS) data in cardiac tissue^7^. Oxidative modification of Trp leads to three kinds of oxidized residues: oxindolyl alanine or 2-oxy Trp (with increased mass of +16 Da over Trp), N-formyl kynurenine (+32 Da) and kynurenine (+4 Da). Screening for Trp oxidation events in the MS data from the CTX model and muscle disease studies^2,3^ revealed several mitochondrial proteins with oxidized Trp, including Aconitase, Voltage-dependent Anion Channels (VDAC), and subunits of mitochondrial complexes I, III and V among others, indicating that Trp oxidation might contribute to altered mitochondrial dynamics.

Proteomics data in the CTX model^2^ revealed that Ubiquinol-Cytochrome C Reductase Core protein 1 (UQCRC1; PDB Id: 1SQB), a subunit of Ubiquinol-Cytochrome C Reductase Complex or Cytochrome b-c1 Complex or mitochondrial Respiratory Complex III (CIII)^8^ contained one oxidized Trp395 (W395; +16 Da). Molecular modelling of UQCRC1 revealed that oxidized W395 could potentially cause a steric clash with the nearby L392. Since we observed lowered enzyme activity of CIII both in the CTX model and human MDs^2,3^, we hypothesize that oxidation of W395 could potentially contribute to altered local conformation which may subsequently impinge on the structure of the complex and enzyme activity. To address this, we performed molecular dynamics simulation studies on the entire CIII complex and revealed that a single modification on W395 in the core protein of CIII causes significant structural changes which may be responsible for hampering CIII function. In order to account for all the structural scenarios of CIII organization, we investigated oxidation-dependent structural changes in inhibitor-bound, substrate-bound, and unbound (apo-form) states of CIII.

## Results and Discussion

**Figure 1:**
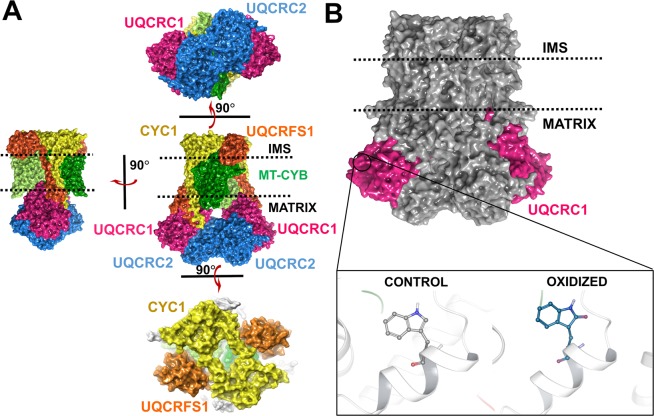
Orientation of Complex III (CIII) (1SQB) on the inner mitochondrial membrane and the location of the post translational oxidation W395. (**A**) – (Central panel) The membrane embedded CIII showing the pivotal subunits involved in electron transfer; (Left panel) Side-view of the right angle rotated, surface represented CIII indicating membrane spanning subunits, UQCRFS1 (orange), CYC (yellow) and MT-CYB (light green and dark green) subunits. The UQCRFS1 and the CYC1 globular domains exist on the intermembrane space (IMS) face of the membrane. The core subunits, UQCRC1 (pink) and UQCRC2 (blue), face the mitochondrial matrix region; (Upper panel) Bottom-view of the right angle rotated, surface represented CIII showing the bilobed structure formed by the core subunits, UQCRC1 and UQCRC; (Bottom panel) Top-view of the right angle rotated, surface represented, CIII showing the arrangement of UQCRFS1 and CYC1, on the IMS facing region. (**B**) – The post-translational oxidation occurs at W395 position of UQCRC1 (pink). The residue is oxidized to oxindolylalanine (+16 Da) form of oxidized Trp. The residues are indicated by ball and stick representation. White sticks represent the carbon atoms of the unmodified residue, blue sticks represent the carbon atoms of the oxidized residue and dark blue and red atoms commonly indicate nitrogen and oxygen atoms, respectively.

CIII is composed of 11 subunits^8–10^ that are arranged as a dimer, embedded in the inner mitochondrial membrane (Fig. 1A). Among these, ten are nuclear encoded, while one is mitochondrial encoded (Table 1). Apart from the core proteins, UQCRC1 and UQCRC2, and the core embedded subunit UQCRFS1, all the other subunits have transmembrane domains. Each subunit of CIII has a specific function that contributes to the electron transfer process of the complex. There exists crosstalk among the CIII subunits, which integrate transfer of electrons from ubiquinone to cytochrome C and proton pumping from the mitochondrial matrix into the inter-membrane space. CIII is also probably involved in mitochondrial precursor peptidase (MPP) activity and superoxide generation^11^, although these require experimental validation *in vivo*. Our recent studies in the mouse model of myodegeneration revealed lowered CIII activity and oxidation of Trp395 (to oxindolylalanine) in UQCRC1^2,3^. UQCRC1 and UQCRC2 have a bilobed structure with a hollow inner core exposed to the matrix. The membrane spanning region of CIII consists of cytochrome b (MT-CYB), ubiquinone-binding protein (UQCRQ), 7.2 kDa transmembrane protein (UQCR10) and 6.4 kDa transmembrane protein (UQCR11) subunits, in addition to the tail region of the iron-sulfur cluster containing protein (UQCRFS1) and cytochrome c1 (CYC1).

**Table 1:** Details of CIII subunits based on the published structure (PDB Id: 1SQB^13^).

Subunit	Gene Name	Chain Name	Sequence length
Ubiquinol-Cytochrome C Reductase Core protein 1	UQCRC1	A/L	480
Ubiquinol-Cytochrome C Reductase Core protein 2	UQCRC2	B/M	453
Mitochondrial encoded Cytochrome b	MT-CYB	C/N	379
Cytochrome c1	CYC1	D/O	241
Ubiquinol-Cytochrome C Reductase Iron-sulfur subunit	UQCRFS1	E/P	196
Ubiquinol-Cytochrome C Reductase binding protein (14 kDa protein)	UQCRB	F/Q	110
Ubiquinol-Cytochrome C Reductase subunit VII or Ubiquinone-binding protein QP-C	UQCRQ	G/R	81
Ubiquinol-Cytochrome C Reductase Hinge protein (11 kDa protein)	UQCRH	H/S	78
8 kDa protein	UQCRFS1	I/T	78
Ubiquinol-Cytochrome C Reductase Subunit 10 (7.2 kDa protein)	UQCR10	J/U	62
Ubiquinol-Cytochrome C Reductase Subunit 11 (6.4 kDa protein)	UQCR11	K/V	56

Through computational studies, we investigated the structural effects of Trp395 oxidation on the entire complex (Fig. 1B), in the inhibitor-bound, substrate-bound, and unbound/apo-form states. All the structures selected for the analysis of the three states were significantly indifferent from each other. The molecular dynamics simulation (MDS) was carried out for 50 ns, using Desmond^12^. The CIII structure of all three states [inhibitor-bound form (PDB ID: 1SQB^13^), substrate-bound form (PDB ID: 1NTZ^14^) and apo-form (PDB ID: 1NTM^14^)], in which Trp395 was modified to oxindolylalanine, was subjected to MDS and compared with the MDS of the corresponding unmodified CIII (control). Detailed structural calculations were performed to analyze the inter-subunit contacts and changes in the secondary structures. Description of the methods and data are provided for 1SQB and 1NTM, while data analysis of 1NTZ is not shown.

### Interpretation of backbone related parameters

The structural parameters investigated in this study are represented by root-mean square deviation (RMSD), root-mean square fluctuation (RMSF) and radius of gyration (Rg), in addition to visual inspection of trajectory data. The RMSD of the oxidized CIII and the constituent structures exhibited significant differences through the simulation time of 50 ns, compared with control. The backbone of the oxidized CIII exhibited higher RMSD from 25 ns, whereas the control backbone of unmodified CIII retained the stability throughout the simulation (Fig. S1A).

The subunit-wise assessment revealed significant RMSF in MT-CYB, CYC1, and UQCRFS1 in the oxidized form compared to control. On the other hand, slight variations in RMSF were observed in UQCRQ, UQCR10 AND UQCR11 (described below) (Fig. S1B). The overall backbone of CIII showed substantial fluctuation of Rg throughout the simulation (Fig. S1C). The Rg data was also calculated for the control and oxidized forms based on the secondary structure.

Most of the α-helices are present in the transmembrane region of CIII, while fewer are in the core subunits UQCRC1 and UQCRC2. Major part of the β-sheets is positioned in the matrix and intermembrane region of CIII. The Rg values of α-helices were rel*a*tively higher in the oxidized form compared to the control (Fig. S1D). However, the relaxation pattern of α-helices followed the same pattern in both. The Rg values for the β-strands were relatively higher in the oxidized form compared to control (Fig. S1E), suggesting a decrease in the compactness in oxidized CIII. Consequently, the structure of CYC1 and UQCRFS1 subunits could be affected, since β-sheets form the significant part of these subunits. The Rg data for the loops showed high fluctuation both in control and oxidized CIII, which may predominantly contribute to the high fluctuation of Rg data of the entire protein backbone of CIII in both control and oxidized forms (Fig. S1C).

### Structural changes in CIII subunits

#### UQCRC1 and UQCRC2

**Figure 2:**
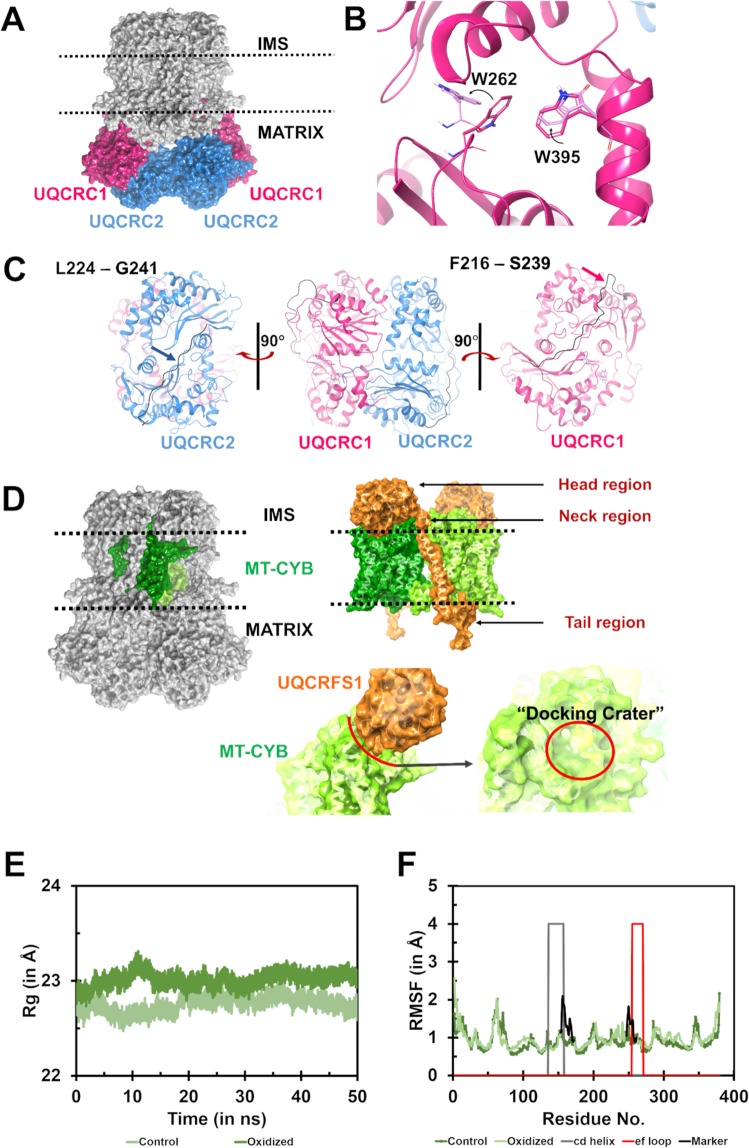
Structural effects exhibited in the subunits UQCRC1, UQCRC2 and MT-CYB. **(A**) – The surface representation of the subunits UQCRC1 (pink) and UQCRC2 (blue) and their location in CIII, embedded on the membrane. The UQCRC1 and UQCRC2 face the matrix region. (**B**) – The side chain conformations of W262 is found to vary in the control (dark pink) and oxidized (light pink) states. (**C**) – (Centre panel) The bilobed structure of UQCRC1 and UQCRC2. The arrows (pink and blue) indicate the loop, which shows higher deviation in the oxidized form (black) compared to the control structure (dark pink). (Right panel) The loop containing the residues F216-S239, running through the surface of the subunit, shows a higher deviation in the oxidized form (black tube) compared to the control (pink tube). (Left panel) Similar to UQCRC1, UQCRC2 also shows a loop containing residues L224-G241, running on the surface of the subunit, which shows higher deviation in the oxidized form (black tube) compared to control (blue tube). (**D**) – Surface representation of the orientation of the transmembrane subunit MT-CYB (green), on the inner mitochondrial membrane. The head domain of UQCRFS1 (orange) lies on the IMS facing side of the membrane. The globular domain has been studied to be docked and undocked as a part of the physiological process of transferring electrons in CIII. The anterior surface of MT-CYB forms the docking crater by rearrangement of the cd helix and ef loop on MT-CYB. (**E**) – Rg analysis of MT-CYB indicates the introduction of compactness in the MT-CYB domain in the oxidized state (light green) as compared to the control state (dark green). The Rg data is measured in Å. F – RMSF analysis shows increased fluctuation in the residues spanning in and around the cd helix (grey marked zone) and ef loops (red marked zone) in the oxidized form (black marker).

UQCRC1 resides on the matrix face of CIII (Fig. 2A) with the oxidized W395 reported in our previous studies^3,5^ lying on a short helix perpendicular to the membrane. This residue lies on a helix which is a part of the continuous helix-loop-helix structure made up by five helices constituted in-between the β-sheet fold. The sequence alignment of UQCRC1 from three mammalian species (bovine, human, and mouse) revealed that Trp395 and the surrounding residues are conserved across species (Fig. S2). MDS data revealed altered conformation of the side-chains of oxidized W395 and the neighboring residue W262, in the oxidized form (Fig. 2B). These conformational changes may significantly alter the neighboring loop structure, which is exposed to the solvent region (Fig. 2C). The oxidation of W395 alters its side chain conformation, thereby preventing hydrophilic interactions with W262. The RMSD values of the backbone atoms of the oxidized form of UQCRC1 were elevated post the initial 5 ns of simulation (Fig. S3A). These values remained unchanged at ~2 Å for the most part of the simulation time (until ~45 ns). Although there was a minor dip, the higher RMSD value was maintained until the end of the simulation.

UQCRC2 complexes with UQCRC1 and participates in MPP activity (although it requires experimental validation *in vivo*) during the processing and assembly of CIII^15^. RMSD values of UQCRC2 did not reveal any significant differences between the oxidized and control CIII (Fig. S3B). The consolidated analyses of RMSD (Figs S3A and S3B), Rg (Fig. S5A), and RMSF (Fig. S5B) corresponding to the UQCRC1 and UQCRC2 and UQCRC2 do not show any significant changes between oxidized and control forms.

#### MT-CYB

This subunit forms the major bulk of the transmembrane region of CIII and houses two potential electron-transferring heme groups (marked b_H_ and b_L_) positioned on the matrix side and inter-membrane side, respectively. MT-CYB possesses a “docking crater” (Fig. 2D) on its intermembrane surface that enables accommodation of the head domain of UQCRFS1 during electron transfer^15^. MT-CYB subunit is also critical for the proton pumping function^16^.

The Rg data indicates an increase in the girth of MT-CYB to maxima of the collected dataset at ~10 ns of the simulation (Fig. 2E). Although the Rg values drop post 10 ns, they are relatively higher compared to the control. The Rg values of MT-CYB are elevated to accommodate the head domain of UQCRFS1, which pushes inward into the “docking crater” on MT-CYB. The RMSF data shows an increase in the per-residue fluctuation in and around the cd helix and ef loop of MT-CYB in oxidized CIII (Fig. 2F), thereby indicating their involvement in MT-CYB structure under the oxidized conditions. The RMSD of the MT-CYB backbone exhibits an increase in the initial 10 ns (vs. control), after which it was stable (Fig. S3C). The Rg analysis correlates well with the RMSF data which shows an increase in the fluctuation near the cd helix of MT-CYB (Figs 2E and 2F).

A similar analysis was performed on the apo-form (PDB ID: 1NTM^14^) and the substrate-bound state of CIII (PDB ID: 1NTZ^14^; data not shown). For the apo-form, the Rg data exhibits reduced the radius of gyration in both the control and the oxidized state, which may be due to the absence of substrate (Fig. S6A). Although the overall increase in RMSF adds proof to the binding site variation, the specific pattern of differences in the per-residue fluctuation in control and oxidized states, near the cd helix and ef loops in 1NTM, showed consistency with the inhibitor-bound structure (1SQB) analysis (Fig. S6B). A similar consistency in the structural differences between the oxidized and unmodified forms was also observed in the substrate-bound form (1NTZ; data not shown).

#### UQCRFS1

**Figure 3:**
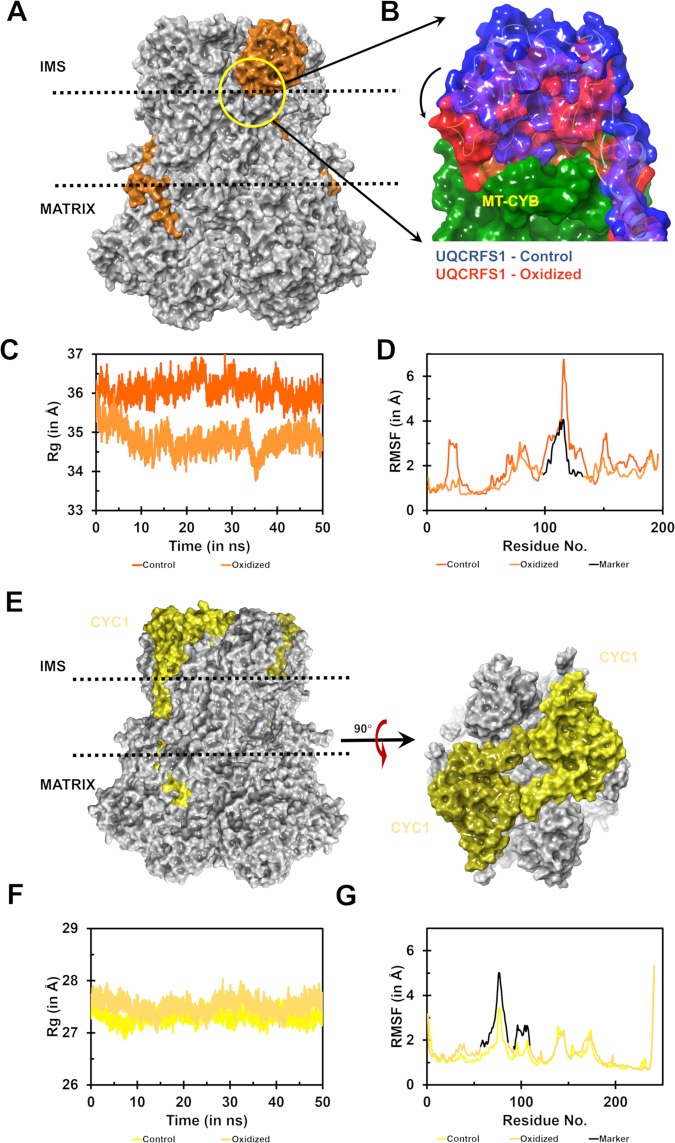
Structural effects exhibited in the subunits UQCRFS1 and CYC1. (**A**) – Surface representation of UQCRFS1 (orange) as a part of CIII, embedded in the inner mitochondrial membrane. (**B**) – Surface representation of the orientation of head domain of UQCRFS1 in control (blue) and oxidized (red) states. It is observed that the head domain docks itself onto the docking crater on the MT-CYB (green). (**C**) – Rg analysis of UQCRFS1 indicates increased stiffness in the oxidized (light orange) state, compared to control (dark orange) state. The Rg calculated is in Å. **D –** RMSF analysis indicates that a part of the globular head of UQCRFS1 (black marker) exhibits reduced fluctuation, indicating its contribution to the compaction of the subunit. (**E**) - Surface representation of CYC1 (yellow) subunit as a part of CIII, embedded in the inner mitochondrial membrane. The top view of the right angle rotated along the membrane axis shows the arrangement of CYC1 on the IMS facing side of the membrane. (**F**) – Rg analysis of CYC1 indicates loosening up of the subunit in the oxidized state (light yellow) compared to the control state (dark yellow). **G** – RMSF analysis shows that the left lateral region of the CYC1 head domain shows higher fluctuation in the oxidized (dark yellow) state as compared to the control (bright yellow) state.

This subunit has highly flexible unstructured loops and few β strands which arrange themselves as β-meanders or β-helix-β super-secondary structures. The oxidized CIII form revealed significant changes in UQCRFS1 structure (Fig. 3A,B), with lowered Rg values for the oxidized structure compared to control (Fig. 3C). RMSF analysis suggested that the fluctuations in the oxidized form are significantly lowered (Fig. 3D). Intriguingly, the loop connecting S107-E131, which is positioned on the left lateral region of the head domain of UQCRFS1, moves inwards post-oxidation (not shown). It occurs due to increased compactness of the head domain accompanied by elevated rigidity of the subunit. Moreover, the neck region, connecting the head domain and the transmembrane tail region is relatively widened compared to control. RMSD analysis shows relatively higher stability, right from the initial stage extending throughout the simulation (Fig. S3D). This data substantiates the fixed nature of UQCRFS1 post oxidation. Angle calculation at the neck region provided evidence for increased stiffness of the mobile neck region of UQCRFS1 (Fig. S5C). Rg analysis of the apo-structure (1NTM) revealed a similar pattern of variation between the control and oxidized forms (Fig. S7A). The RMSF analysis of the apo-structure also exhibited similar restriction pattern of the loop region, as seen in 1SQB (Fig. S7B). A similar trend was also noted in the substrate-bound form (1NTZ; not shown).

#### CYC1

CYC1 is the destination subunit that receives electrons via the bifurcated mechanism and passes them to the extrinsically bound Cytochrome C. Cytochrome C, in turn, detaches and carries the electrons to the next complex of the respiratory chain. CYC1 forms an integral part of CIII because of its dominant presence in the inter-membrane space along with UQCRFS1 (Fig. 3E). CYC1 possesses a globular head domain that interacts with UQCRFS1 head region on the matrix exposed face and extends its tail domain which spans the membrane once before it overlays on UQCRC1 through charged interactions as discussed below. Rg analysis showed relatively high values throughout the simulation in the oxidized form which suggests increased girth of CYC1 (Fig. 3F). The RMSD data indicates increased instability of the oxidized form post 35 ns compared to control (Fig. S4A). The RMSF data revealed that the loop extending from T57 to K86 and P92 to L109 had increased fluctuation in the post oxidation state (Fig. 3G). These alterations correlated well with Rg and RMSD data. Further, it has direct implications for electron transfer since the region 63–81 contribute to the binding with cytochrome C^17^, which may be affected in the oxidized form (Fig. S5D). Rg analysis showed higher values for the oxidized state than the control (Fig. S7C). The apo-structure (1NTM) revealed a similar pattern of variation as in 1SQB. RMSF analysis of the CYC1 domain in the apo-form also revealed the distortion at cytochrome C binding site (Fig. S7D). A similar trend was also noted in the substrate-bound form (1NTZ; not shown).

### The UQCRC1 Interactors

#### Interactions between UQCRC1 and UQCRC2/CYC1

**Figure 4:**
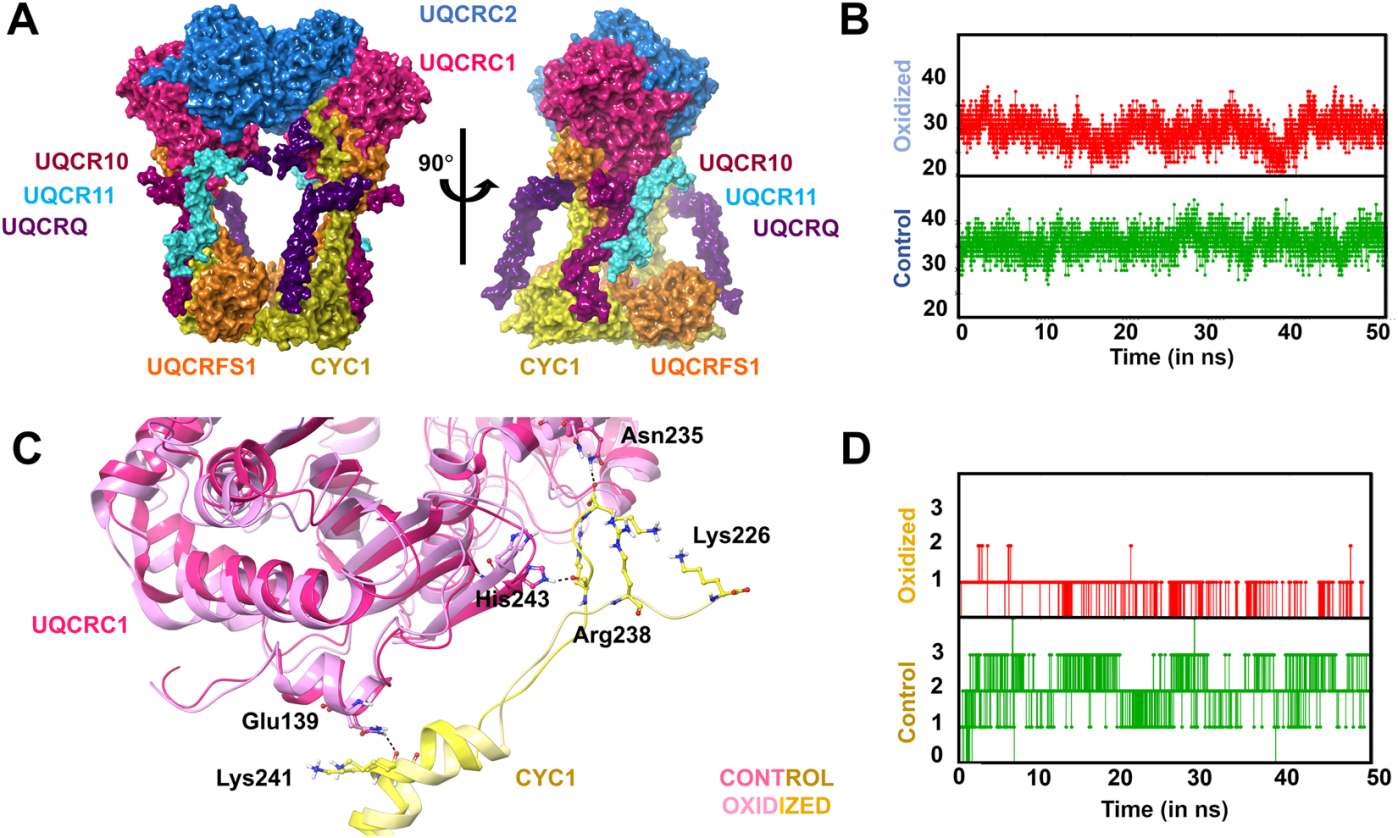
Interactors of UQCRC1 – UQCRC2 and CYC1. (**A**) – Surface represented UQCRC1 (pink) is shown to have tangible interactions with UQCRC2 (blue), CYC (yellow), UQCRFS1 (orange), UQCRQ (violet), UQCR10 (purple) and UQCR11 (cyan). (**B**) – H-bond analysis between UQCRC1 and UQCRC2 shows ~10 bond decrease in the oxidized form (red) compared to control (green) state. (**C**) – Interaction between the C-terminal regions of the CYC1 with UQCRC1 is mediated by Glu139, His243 and Asn235 in the former (represented in yellow sticks) with the residues Lys241, Arg238 and Lys226 in the latter (represented in pink sticks). Post-oxidation, the residues Arg238 and Lys226 of CYC1 were found to drift away from UQCRC1 breaking the bonds. (**D**) - H-bond analysis between UQCRC1 and CYC1 shows two-thirds decrease in the hydrogen bond contacts between the subunits explained due to loss of interactions as in C.

UQCRC1 interacts directly with UQCRC2, UQCRFS1, CYC1, UQCRQ, UQCR10 and UQCR11 (Fig. 4A). The N-terminal of all these subunits except CYC1 interacts with the solvent accessible surface of UQCRC1. The C-terminal of CYC1 interacts with the UQCRC1 domain on the matrix region. Hydrogen bond analysis revealed that the individual contacts established by UQCRC1 with UQCRFS1, UQCRQ and UQCR11 were relatively increased in the oxidized state compared to the control (described below). The hydrogen bond contacts of UQCRC1 with UQCRC2, CYC1, and UQCR10, respectively have decreased drastically in the oxidized CIII form (described below).

UQCRC1 makes a stable complex with UQCRC2 through extensive intermolecular hydrophilic interactions. A closer look into the hydrogen bond interactions at the UQCRC1-UQCRC2 interface reveals a hemispherical sealing effect (not shown). Interestingly, the number of hydrogen bonds was significantly reduced in the oxidized form compared to control (Fig. 4B). It was associated with loss of interactions between the N-terminal flexible region of UQCRC1 and UQCRC2, which in turn directs the movement of the helix preceding the N-terminal tail in UQCRC1 to push away from UQCRC2 (not shown).

The C-terminal region of CYC1 interacts with the matrix-facing globular domain of UQCRC1 (Fig. 4A). The residues, Asn235, His243 and Glu139 in UQCRC1 were found to interact with Lys226, Arg238 and Lys241, respectively, in unmodified CIII (Fig. 4C). Hydrogen-bond analysis between UQCRC1 and CYC1 showed decreased hydrogen bonds from three to one in the oxidized form **(**Fig. 4D**)**. The interaction between the side chain of Glu139 (UQCRC1) and Lys241 (CYC1) was sustained both in the oxidized form and control. However, the loss of contacts is at the C-terminal unstructured region of CYC1 where Arg238 and Lys226 interact with His243 and Asn235 of UQCRC1, respectively. It occurs in coordination with the repulsion of the C-terminal of CYC1 away from UQCRC1 **(**Fig. 4C**)**.

#### Interactions between UQCRC1 and UQCRFS1/UQCRQ

**Figure 5:**
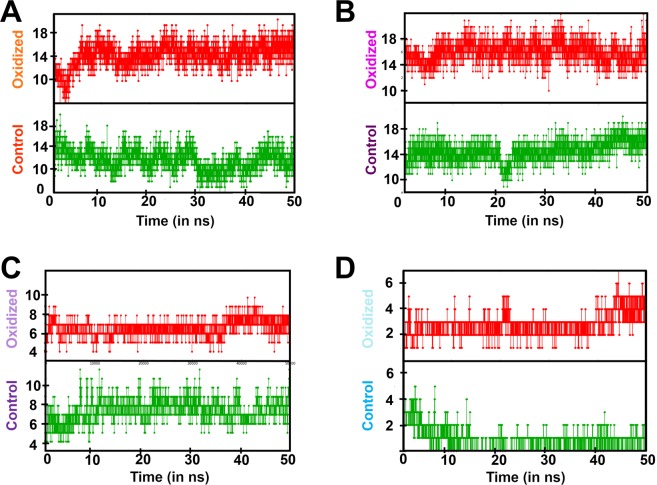
Hydrogen bond analysis between UQCRC1 and the subunits: (**A**) – UQCRFS1 (orange); (**B**) – UQCRQ (violet); (**C**) – UQCR10 (purple); and (**D**) – UQCR11 (cyan). The green panel exhibits the trend in the control state and the red panel exhibits the trend in the oxidized state.

The transmembrane tail domain of UQCRFS1 extends to the matrix-facing region and interacts closely with UQCRC1, whereas the head domain lies on the inter-membrane space away from UQCRC1 (Fig. 4A). The N-terminal of UQCRFS1 interacts with one of the two L-shaped helix-turn-helix motifs of the UQCRC1 on the exterior surface of the latter and lies parallel to the N-terminal of CYC1. The hydrogen bond contacts between the UQCRC1 and UQCRFS1 were increased in the oxidized form compared to the control (Fig. 5A), indicating increased stability of the complex formed between the two subunits.

UQCRQ interacts with UQCRC1, MT-CYB, UQCRFS1, and CYC1 (Fig. 4A). UQCRQ is sandwiched between UQCRC1, CYC1, and UQCRFS1 on the matrix side. The region, H12-S17 on UQCRQ forms alternate backbone hydrogen bonding with UQCRC1 and UQCRFS1. A part of UQCRQ interacts with MT-CYB on the intermembrane side as well as throughout the membrane-spanning region. However, there are no interactions between UQCRQ and CYC1 or UQCRFS1 on the intermembrane side, in control. Hydrogen bond analysis between UQCRC1 and UQCRQ revealed a higher number of hydrogen bonds in the oxidized form compared to the control (Fig. 5B), indicating increased stability between the interacting subunits UQCRC1 and UQCRQ.

#### Interactions between UQCRC1 and UQCR10/UQCR11

UQCR10 is a single pass transmembrane helix with a loop-short helix on the non-membrane spanning region. The short loop of the helix-loop-helix structure on UQCR10 at the N terminal interacts with UQCRC1. The former lies in close proximity to the transmembrane helices of UQCR11 and UQCRFS1 on the matrix-facing side. On the other hand, UQCR10 interacts closely with CYC1 on the intermembrane space facing region (Fig. 4A). This arrangement of UQCR10 may be responsible for transmitting signal from UQCRC1 to other subunits. Hydrogen bond analysis between UQCRC1 and UQCR10 in the oxidized form exhibited a substantial decrease in the number of contacts (Fig. 5C), suggesting weakened stability of the interaction between UQCRC1 and UQCR10.

UQCR11 associates with the neighboring subunits UQCRC1 and UQCR10 (Fig. 4A). The N terminal region of the subunit interacts with UQCRC1 at the interior bottom surface. Hydrogen bond analysis suggested that although the control possesses ~3–4 hydrogen bonds initially, post 5 ns, the average number of hydrogen bonds secured between the two subunits falls to one (Fig. 5D). In the oxidation state, the trend is reversed. There are ~3 hydrogen bonds between the two subunits till 45 ns, which increases to ~5. It may probably cause a stiffening effect in UQCR11. On validation, the consistent hydrogen bond was established between the Gln12 of UQCR11 and Glu351 of UQCRC1. The post-oxidation trajectory revealed additional hydrogen bonds between Trp24 and Asn16 from UQCR11 with Arg445 and Thr347 of UQCRC1, respectively (not shown).

### Other Interactions

#### Interaction between UQCRFS1 and CYC1

**Figure 6:**
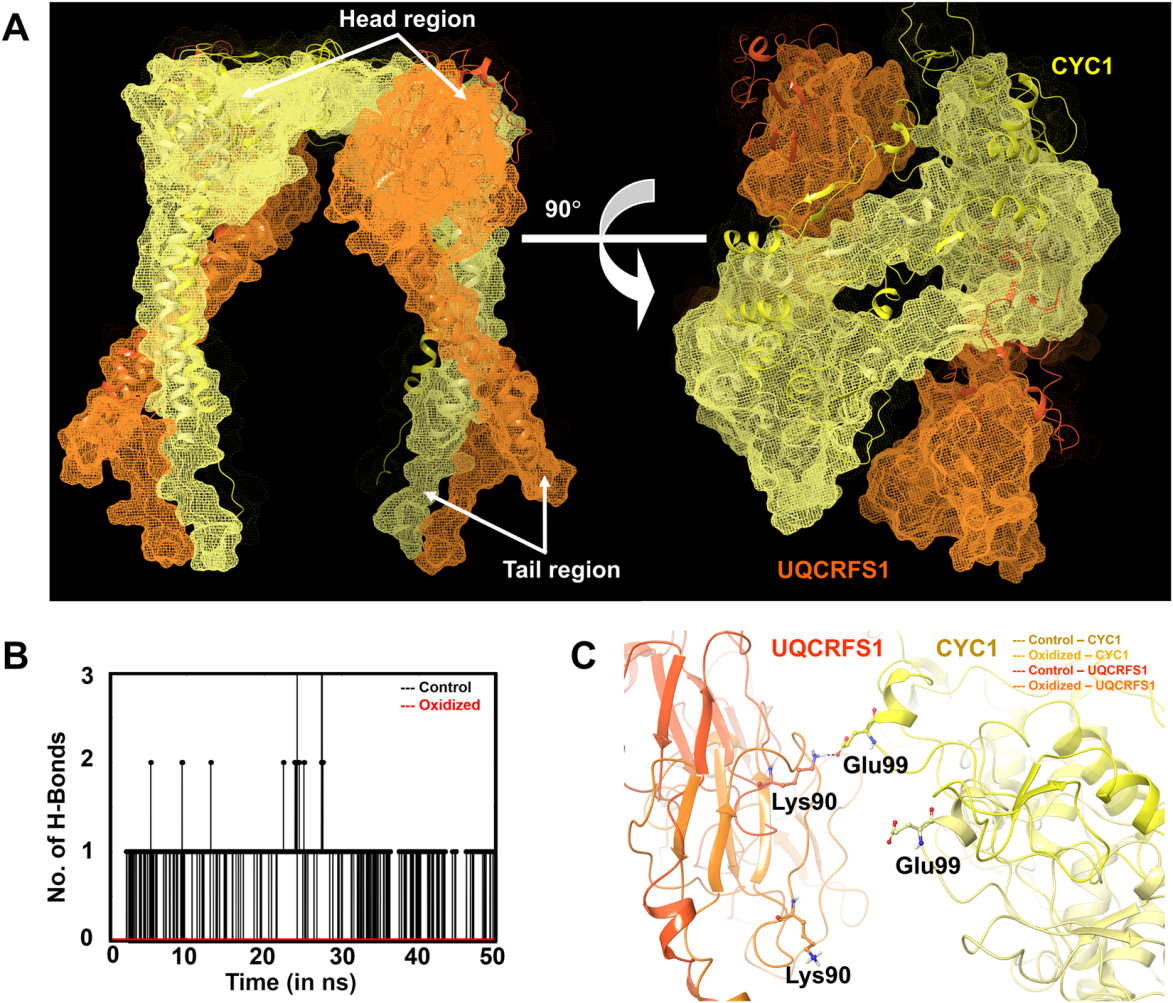
Interaction between UQCRFS1 and CYC1. **(A**) – (Left panel) Front-view of the mesh representing UQCRFS1 (orange) and CYC1 (yellow) embedded to the inner mitochondrial membrane. (Right panel) Top-view of the right angle rotated CIII along the membrane plane. (**B**) – H-bond analysis between UQCRFS1 and CYC1 shows abolishment of the single hydrogen bond contact in the oxidized form (red) compared to control (black). (**C**) – Oxidation causes a drift in between the residues Lys90 of UQCRFS1 (shown in light orange sticks) and Glu99 of CYC1 (shown in dull yellow sticks). They interact consistently throughout the simulation in the control state (shown in dark orange and bright yellow sticks for Lys90 and Glu99, respectively).

UQCRFS1 and CYC1 are embedded in the membrane, with its globular heads facing the intermembrane space and the tail regions exposed to the matrix (Fig. 4A). Their respective tails interact with UQCRC1 at the matrix-facing region. The head domain of CYC1 of one monomer interacts with the head domain of UQCRFS1 of the other monomer of the dimer (Fig. 6A). This crisscross link establishes CIII as a functional monomer, although it exists as a structural dimer^11^. An intermolecular hydrogen bond interaction between Lys90 of UQCRFS1 and Glu99 of CYC1 was present, in control throughout the simulation, whereas it was completely lost in the oxidized form (Fig. 6B). We speculate from this analysis that the loss of interaction at the head region of UQCRFS1 may lead to a significant shift of UQCRFS1 with respect to CYC1, probably due to a hinge movement at the neck region (Fig. 6C).

#### Distance between the electron transferring heme groups and iron-sulfur clusters

**Figure 7:**
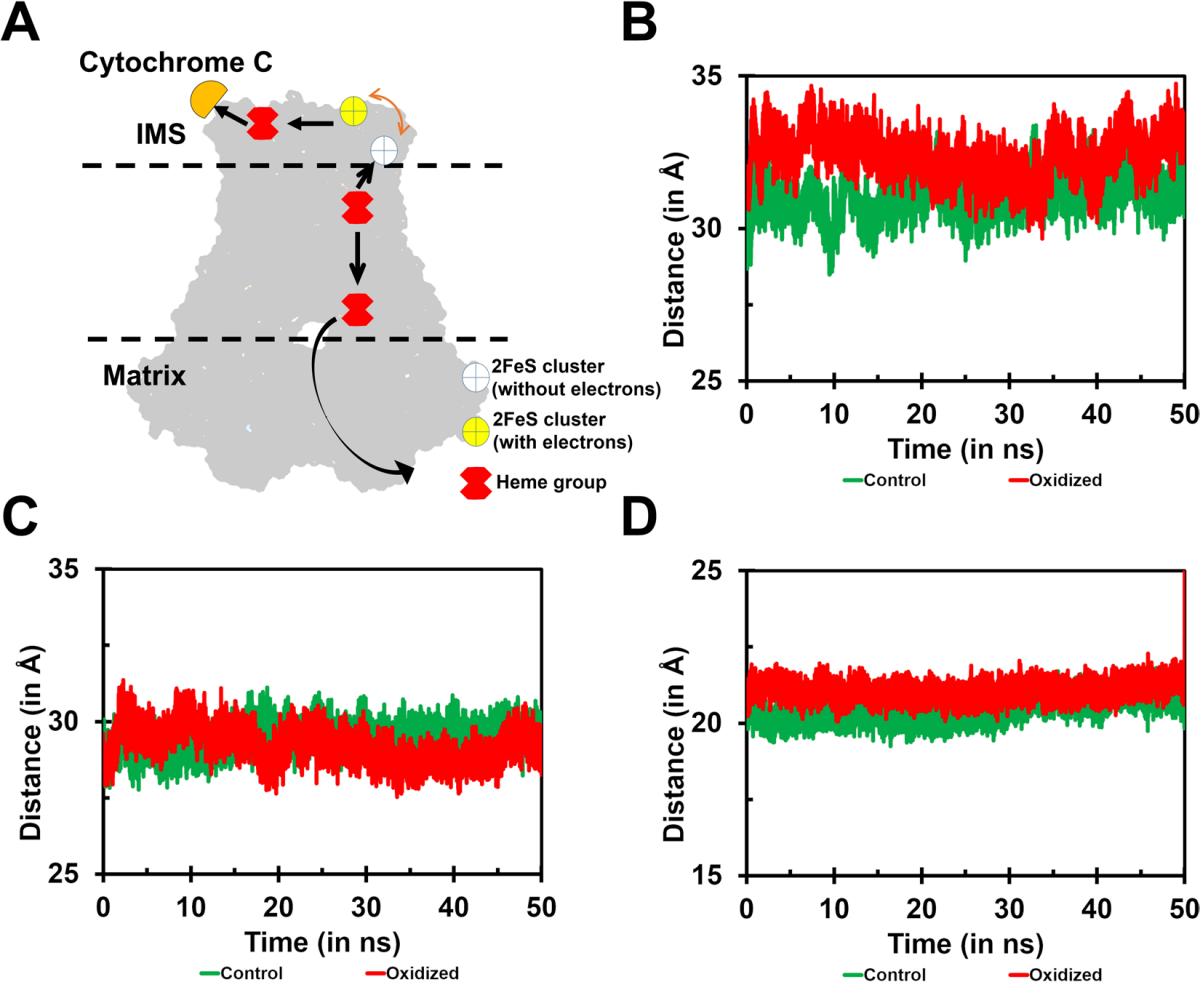
Distance analysis between Electron transfer groups. (**A**) – Schematic diagram indicating the electron flow between the transfer groups in CIII. The electron flow shows a bifurcated system from b_H_ to b_L_ and 2[Fe-S] in two opposite directions. The disruption of distance range within any of these limits causes a break in the electron flow. (**B**) – Distance analysis between the two heme groups housed in the MT-CYB subunit. (**C**) – Distance analysis between the 2[Fe-S] of UQCRFS1 and heme group from CYC1. (**D**) - Distance analysis between the b_H_ of MT-CYB subunit and 2[Fe-S] of UQCRFS1.

The MT-CYB contains two heme groups that play a critical role in electron transfer (Fig. 7A). Distance analysis between these heme groups indicated a slight increase in the oxidized form (Fig. 7B). However, the average distance of these two hemes in the oxidized form is relatively stable compared to the control. The increase in the distance between the iron atoms of the hemes correlates well with the higher Rg in the oxidized form as shown previously (Fig. 2E). The subunits UQCRFS1 and CYC1 contain one iron-sulfur cluster and one heme group, respectively. They complete the line of the electron transfer process to the transporter protein, cytochrome C. Interestingly, the distance analysis between these two cofactors indicated a fluctuation on the higher side in the oxidized form compared to the control (Fig. 7C). The distance between the iron-sulfur cluster in the UQCRFS1 and heme (b_H_) in MT-CYB reaches its minima around 20 ns of simulation time and retains the position for a while, although the distance was not comparable to the range observed, in control (Fig. 7D). It suggests that the head domain of UQCRFS1 probably moves further towards MT-CYB in the oxidized form. Distance analysis performed between the electron transfer groups of the apo structure (1NTM) showed a similar range of intermolecular distances (Figs S8A and S8C) except the distance between the 2[Fe-S] cluster of UQCRFS1 and the heme group from CYC1, which showed significant decrease in the distance of the oxidized form compared to the control state (Fig. S8B). It may be due to the increased flexibility of the head domain of the UQCRFS1, speculating a possibility of occupying the docking crater of MT-CYB in the control state of the apo-structure. For the substrate-bound complex, the distance analyses showed consistent with the inhibitor-bound structure (not shown). Distance analyses revealed that the electron transfer function may be lost due to significant changes in the distance between the clusters, in the oxidized form. This could, in turn, lower the enzyme activity of the complex.

## Conclusion

Mitochondrial respiratory chain complexes have multi-subunit structures that offer a distinct advantage during electron transfer and proton pumping activities. Such intricate structural organization is required for optimal orientation of the Fe-S clusters and other components to improve the overall metabolic efficiency. However, the multi-subunit structure has certain limitations, including vulnerability to PTMs, as exemplified in the current study. During pathophysiological conditions such as myodegeneration, mitochondrial dysfunction, and oxidative damage are evident. Degeneration-dependent protein oxidation could potentially regulate large protein complexes such as CIII.

**Figure 8:**
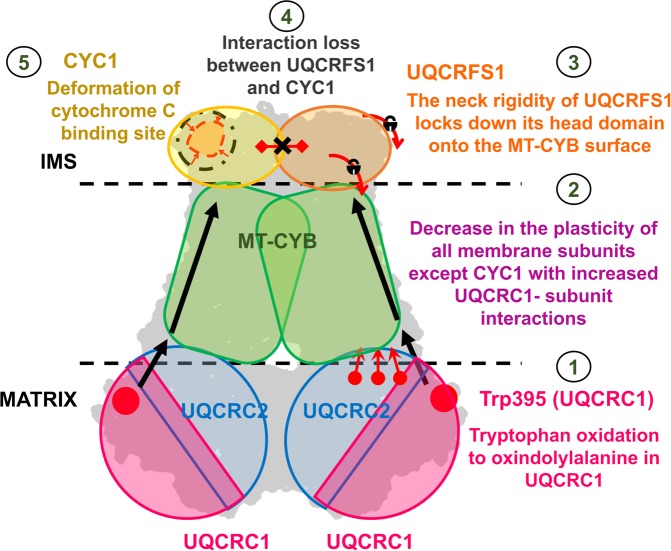
Schematic representation of the structural crosstalk extended throughout CIII due to W395 oxidation in UQCRC1. The sequence of structural effects exerted involved: (1) local conformational changes near W395; (2) decreased plasticity of the membrane-spanning subunits shown with increase in interactions with UQCRC1 and increased Rg in the inhibitor/substrate-bound form and decrease in the apo-form; (3) Introduction of neck rigidity in UQCRFS1 leading to fixation of its head domain; (4) Loss of electron flow between UQCRFS1 and CYC1 because of the lockdown of former subunit; (5) distortion of binding site at CYC1 causing hindrance to the binding of cytochrome C.

Based on molecular dynamics simulations, the current study demonstrated that a single PTM (i.e., W395) in a subunit (UQCRC1) farther from the active site could trigger profound structural changes across the complex thereby disrupting the electron flow and lowering the enzyme activity in CIII (Fig. 8). The reduction in the maintenance of stable hydrogen bonds between UQCRC1 and UQCRC2 dictates the instability of the core domains, thus affecting CIII maturation. Other interactors of UQCRC1 like CYC1 and UQCR10 also showed decreased stability in interactions with UQCRC1. The interactors, UQCRFS1, UQCRQ, and UQCR11 exhibit increased the stability of complex formation with UQCRC1. The functionality of UQCRFS1 depends on the flexibility of the neck region to mobilize its intermembrane space facing the head domain and carry out the electron transfer. Hindrance in this functionality occurs owing to the stiffening of the neck region, causing the fixation of the head domain on the MT-CYB surface. This fixation reduces the distance between the electron transfer groups of UQCRFS1 and CYC1. Additionally, the deformation at the Cytochrome C binding site hinders the prospects of the transfer of electrons, if at all, from CIII to Complex IV. By considering these analyses, we propose that the structural effects of this oxidation directly impinge on electron transfer through the pivotal subunits taking part in the electron transfer process (CYC1 and UQCRFS1)). Further studies may reveal the effects of such pathological post-translational effects on the proton-pumping efficiency through *in-vitro* studies.

## Methods

### Generation of oxidized CIII

The CIII dimer complex containing 22 subunits (PDB Id: 1SQB^13^) embedded with the coordinates of membrane position was downloaded from the OPM (Orientation of Proteins in Membranes) database^18^ and implanted into POPC (phosphatidylcholine) membrane. The structure also contains ligand azoxystrobin, which binds in the Q_0_ site. The oxidized CIII was generated by manipulating the chemical structure of Trp at position 395 in the subunit UQCRC1 to the oxidized state, i.e., oxindolylalanine (with a mass increase of +16 kDa), based on the mass spectrometry studies carried out previously^2,3^. The proteins were processed using the protein preparation wizard module of the Schrodinger Drug Discovery Suite. The protein structures were reviewed for the presence of any important water molecules for the simulation. All the water molecules were deleted before the simulation, considering that none of them established any important bonds with the protein in its vicinity. The ligand, azoxystrobin was retained in the protein structure. Ligand state was generated for pH 7.0. Further, the H-bonds were optimized to the neutral pH, followed by restrained minimization converging the heavy atoms close to 0.30 Angstrom (Å). The same procedure of preparation of oxidized complex and the subsequent methods including MDS and data analysis was followed for the unbound/apo-form (PDB: 1NTM^14^ containing all the 22 subunits) and the substrate-bound CIII (PDB: 1NTZ^14^) without any changes.

### Membrane set-up and relaxation

The POPC membrane was set up on the retrieved prealigned membrane coordinates. The lipid-protein equilibration/relaxation protocol was utilized as prescribed by Desmond membrane relaxation protocol, developed by Dmitry Lupyan in collaboration with Schrodinger Inc (New York, NY, USA)^12^. The relaxation was carried out at a temperature of 300 K. The various steps in this relaxation process involved (i) minimization with restraints on solute atoms (Protein atoms) (ii) minimization without any restraints (iii) heating from 0.0 K to 300.0 K (iv) H_2_O barrier and gradual restraining, followed by (v) NPT (isothermal – isobaric) equilibration with and without the barrier. The NPT ensemble itself consisted of 5 steps in sequential order starting from (i) NPT ensemble with barrier for 200 ps (ii) NPT ensemble equilibration of solvent and lipids for 100 ps (iii) NPT ensemble with protein heavy atoms annealing from 10.0 kcal/mole to 2.0 kcal/mole for 600 ps (iv) NPT ensemble with restraints on C-alpha atoms at 2.0 kcal/mole for ps and finally (v) NPT ensemble with no restraints for 100 ps.

### Molecular dynamics production

The final molecular dynamics production was carried out for a simulation time of 50 ns by allowing the default relaxation of the system at a temperature of 300 K and pressure of 1.01 bar. The trajectory files were recorded for every 4.8 ps. The final simulation trajectories were analyzed using other Desmond operations including the generation of protein-protein interaction data, Root mean square deviation (RMSD), Root mean square fluctuation (RMSF) and radius of gyration (Rg) of the proteins. Following the close visual inspection, calculation of the qualitative, and quantitative data comprising the number of hydrogen bonds, distances, and angle measurements were carried out. PyMOL^19^ was used for viewing trajectories and exhibition of the essential aspects as illustrations.

### Analysis of trajectories

The 50 ns trajectories of the control (unmodified) CIII was analyzed for protein backbone parameters such as the RMSD, RMSF, secondary structure variations and Rg throughout the duration of the simulation. The RMSD and Rg values were calculated against the simulation time and expressed as the deviation or radius of the selected group of atoms, respectively, in Å. The RMSF values of the protein backbone were calculated over the range of residues and expressed as summation throughout the simulation for each residue and were expressed in Å. Although the RMSD and Rg values were calculated for the protein backbone, the same parameters were also calculated for the individual subunits of CIII to describe the detailed effects translated over the timeline.

The trajectories were stripped off from the POPC membrane and water (5.0 Å from the protein surface). The number of hydrogen bond contacts was analyzed between pivotal pair of subunits of CIII involved in electron transport. These contacts were visualized in the trajectory to derive the location and time point of the differences observed between the control and oxidized forms of CIII. The interactions were also supported with evident information of gain or loss of contacts and change in structural conformation through the calculation of angles and distances wherever required. The distances between electron transfer groups involving the heme and iron-sulfur (2[Fe-S]) clusters were also calculated for the control and oxidized form. The Desmond module was used for the calculation of parameters. Maestro and PyMOL were used for the generation of high-resolution illustrations.

### Multiple sequence alignment

The protein sequences of the UQCRC1 subunit of CIII for the eukaryotic species – bovine, murine and human, were retrieved from the Uniprot database^20^. The multiple sequence alignment was performed using Clustal Omega^21^. The alignment was plotted using ESpript^22^.

## Supplementary information

Supplementary Information
