# Supplementary material for: Tryptophan Oxidation in the UQCRC1 Subunit of Mitochondrial Complex III (Ubiquinol-Cytochrome C Reductase) in a Mouse Model of Myodegeneration Causes Large Structural Changes in the Complex: A Molecular Dynamics Simulation Study
Source: Sci Rep. 2019 Jul 23;9:10694. doi: 10.1038/s41598-019-47018-6 (PMC6650490; doi:10.1038/s41598-019-47018-6)
Supplement: Supplementary file 1 — Supplementary Information [file 41598_2019_47018_MOESM1_ESM.pdf]

Tryptophan Oxidation in the UQCRC1 Subunit of Mitochondrial Complex III (Ubiquinol-Cytochrome C Reductase) in a Mouse Model of Myodegeneration Causes Large Structural Changes in the Complex: A Molecular Dynamics Simulation Study

Sruthi Unni[1], S. Thiyagarajan[2], M.M. Srinivas Bharath[3,4*], B. Padmanabhan[1*]

[1]Department of Biophysics, National Institute of Mental Health and Neurosciences (NIMHANS), Hosur Road, Bangalore-560029, Karnataka, India. [2]Institute of Bioinformatics and Applied Biotechnology (IBAB), Biotech Park, Electronic City Phase I, Electronic City, Bangalore- 560100, Karnataka, India. [3]Department of Clinical Psychopharmacology and Neurotoxicology, National Institute of Mental Health and Neurosciences (NIMHANS), [4]Neurotoxicology Laboratory at the Neurobiology Research Center, National Institute of Mental Health and Neurosciences (NIMHANS), Hosur Road, Bangalore-560029, Karnataka, India.

***To whom correspondence should be addressed**

MMSB: bharath@nimhans.ac.in

BP: paddy@nimhans.ac.in

Running title: Trp oxidation induces structural changes in mitochondrial complex III

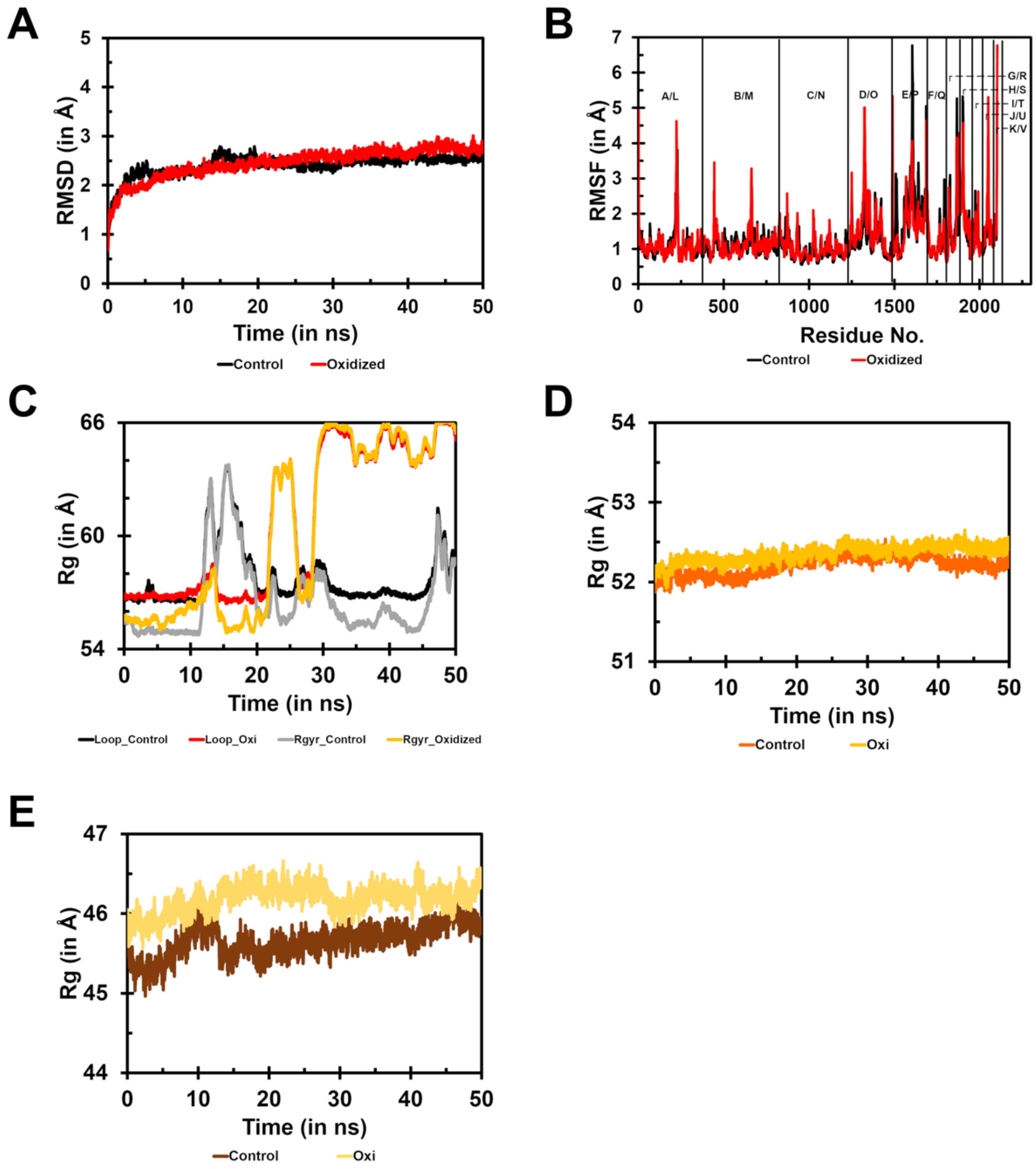

Figure S1 – Structural parameters of the entire Complex III. **A** – RMSD analysis of complete protein backbone of CIII. **B** – RMSF analysis of CIII protein backbone; demarcated for the individual subunits described in Table 1. **C** – Rg analysis of protein backbone of CIII for control (grey) and oxidized (yellow) states. The Rg analysis of overall loops in CIII are also plotted on the same to portray its higher contribution to the overall protein Rg compared to the helices and β-strands (Control – black; Oxidized – red). **D** – Rg analysis of α-helices in CIII (Control – orange; Oxidized – yellow). **E** - Rg analysis of β-strands in CIII (Control – brown; Oxidized – yellow).

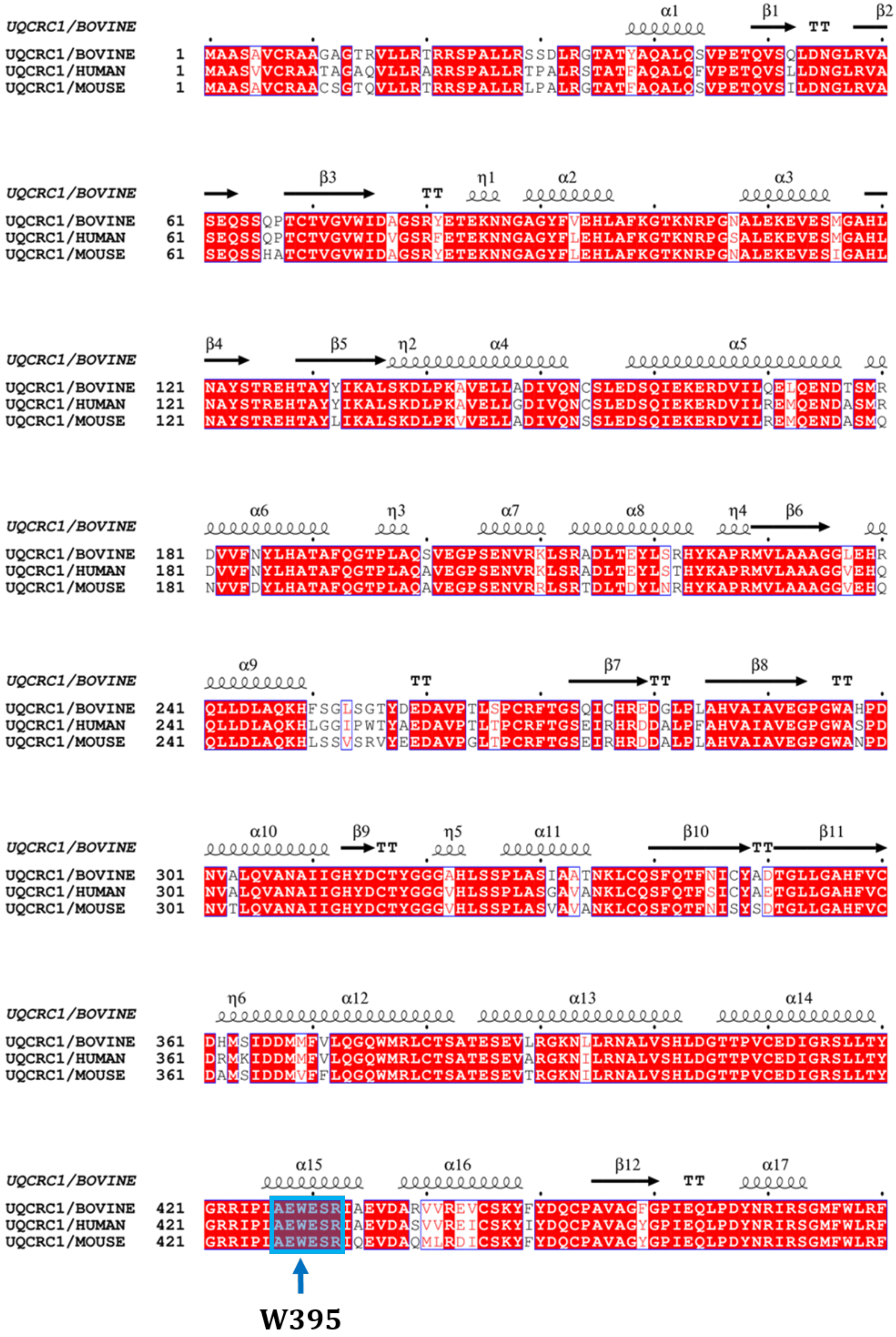
UQCRC1/BOVINE
UQCRC1/HUMAN
UQCRC1/MOUSE
W395

Figure S2 – Sequence comparison of the subunit UQCRC1 of CIII among the mammalian species: bovine, human and mouse. The amino acid sequence alignment is produced by ClustalW (Thompson et al. 1994). Residues with the red background indicate completely conserved amino acids. The oxidized tryptophan, W395, indicated with an arrow, and the neighboring amino acids boxed in cyan are conserved in the three species. The figure is generated by ESpript (Gouet et al. 1999).

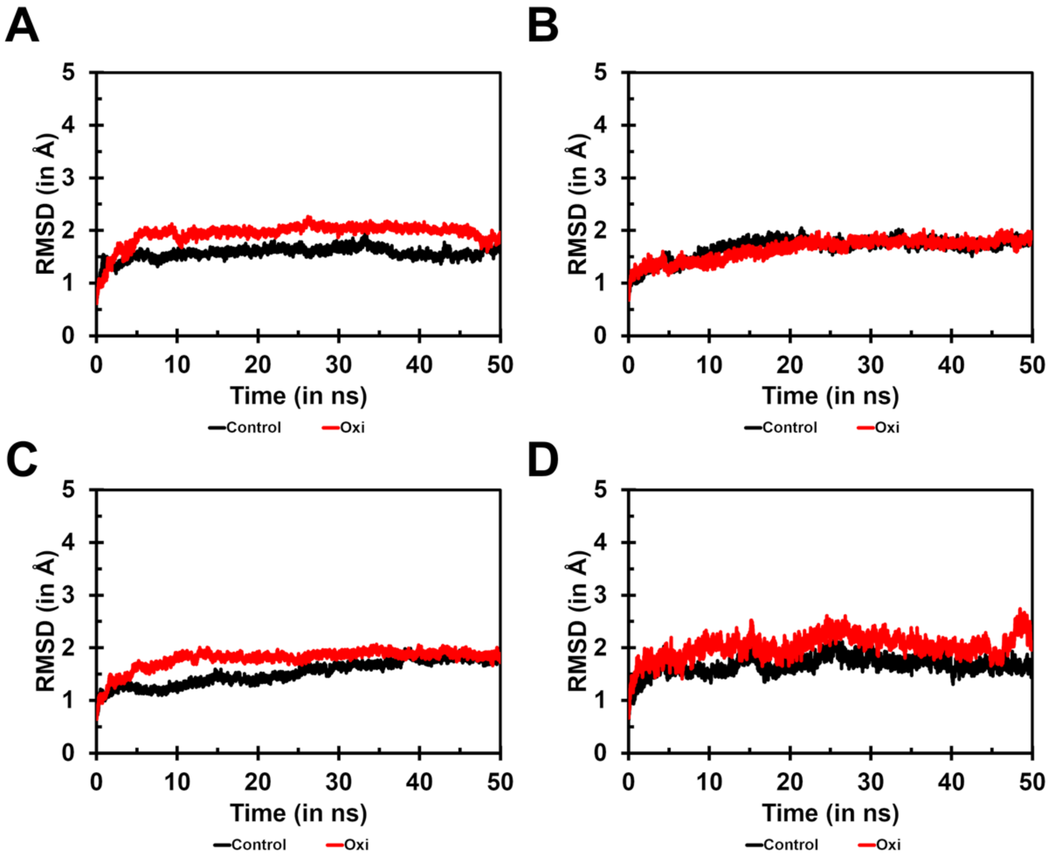

Figure S3 – RMSD analyses for individual subunits. **A** – UQCRC1; **B** – UQCRC2; **C** – MT-CYB; **D** – UQCRFS1. The control and oxidized states are depicted by black and red lines, respectively.

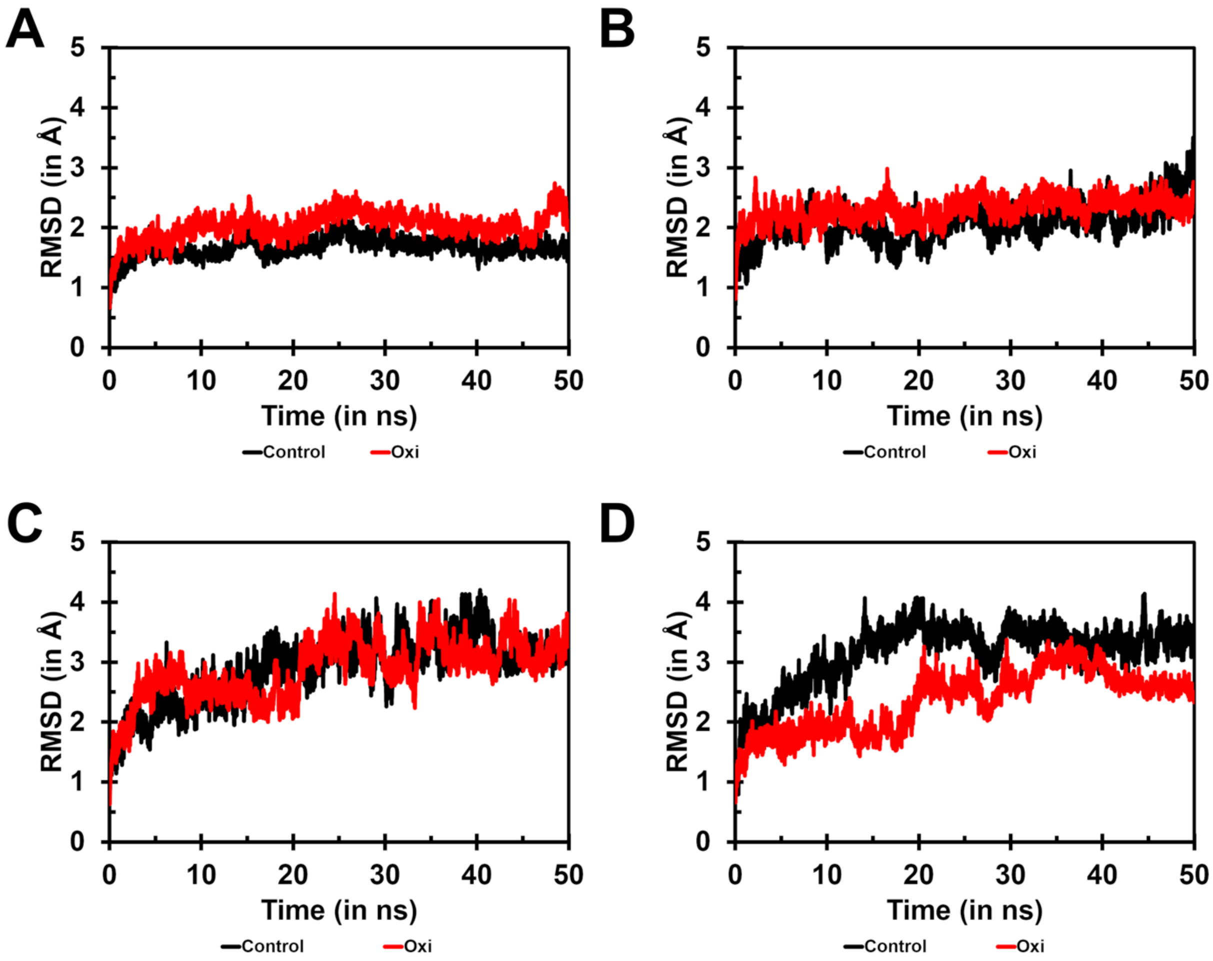

Figure S4 – RMSD analyses for individual subunits (continued). **A** – CYC1; **B** – UQCRQ; **C** – UQCR10; **D** – UQCR11.

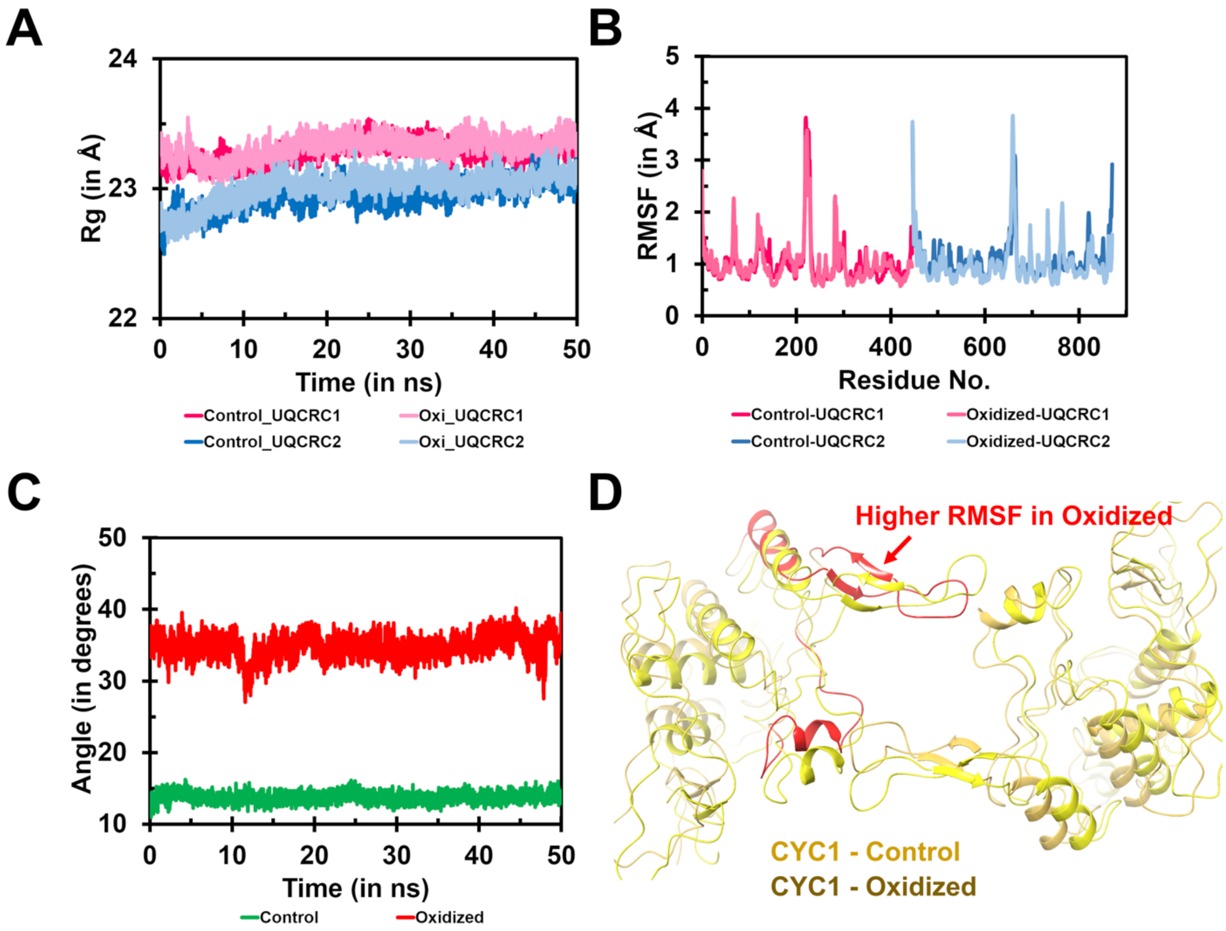

Figure S5 – A – Rg analysis of UQCRC1 (Control – dark pink; Oxidized – light pink) and UQCRC2 (Control – dark blue; Oxidized – light blue). **B** – RMSF analysis for the subunits, UQCRC1 (Control – dark pink; Oxidized – light pink) and UQCRC2 (Control – dark blue; Oxidized – light blue). **C** – Angle calculation at the neck region of UQCRFS1 in control (green) and oxidized (red) states. **D** – Interaction between the two monomers of CYC1 showing increased RMSF fluctuation at the region 63-81 in the oxidized state (red).

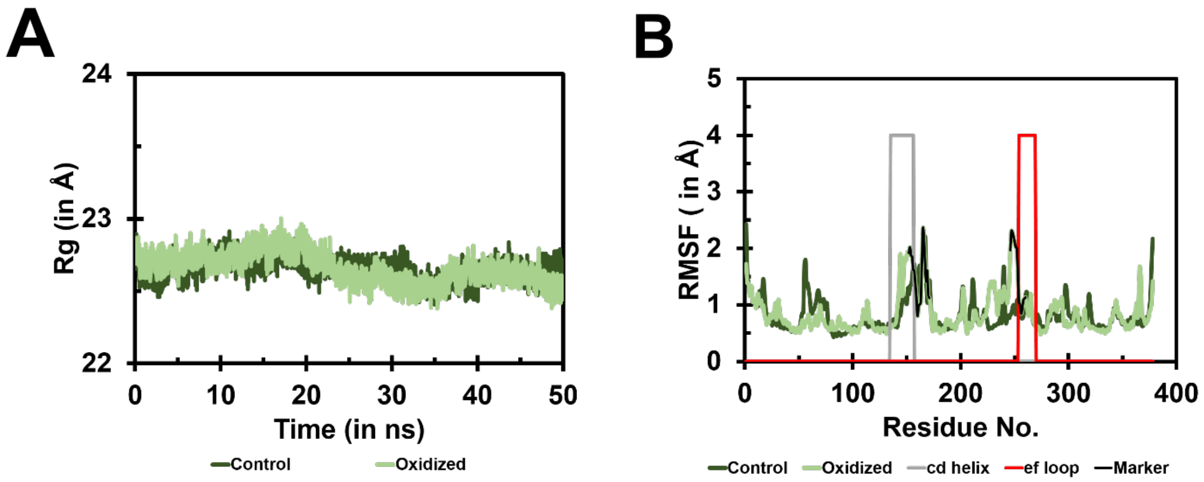

Figure S6 – Structural effects exhibited in the structure of the subunit MT-CYB of CIII apo structure. **A** – Rg analysis (in Å) of apo-form MT-CYB indicates no significant changes in the domain in oxidized state (light green), compared to control state (dark green). **B** – RMSF analysis shows increased fluctuation in the residues spanning in and around the cd helix (area marked by grey box) and the ef loop (area marked by red box) in the oxidized form (black marker).

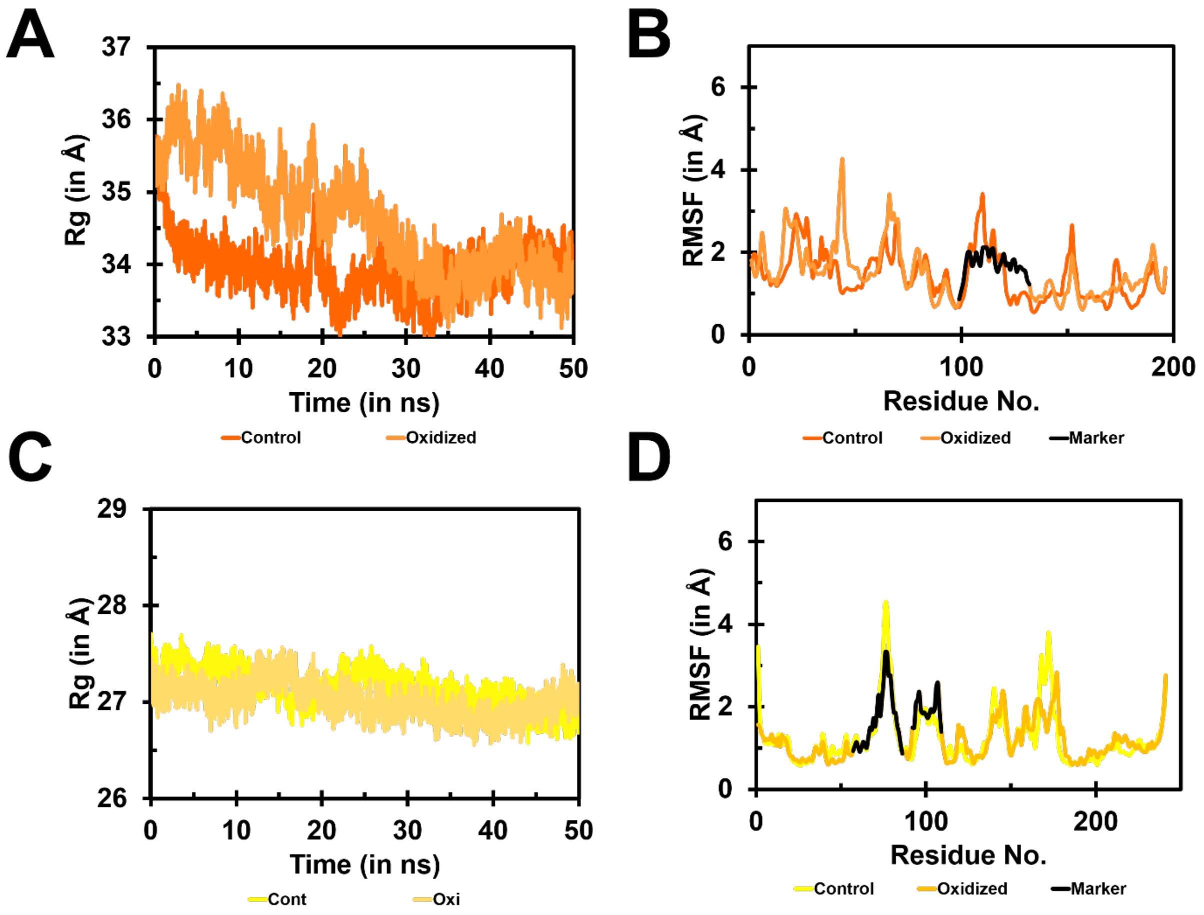

Figure S7 - Structural effects exhibited in the structure of the subunits UQCRFS1 and CYC1 of CIII apo structure. **A** – Rg analysis (in Å) of UQCRFS1 indicates higher Rg values of the oxidized state (light orange), compared to control state (dark orange). The Rg data of both states converged post 30ns. **B** – RMSF analysis shows decreased fluctuation in the residues that form a part of the globular head (black marker) indicating a compaction of that region in UQCRFS1. **C** – Rg analysis (in Å) of CYC1 indicates no significant changes in the oxidized state (dark yellow), compared to control state (bright yellow). **D** – RMSF analysis shows consistently higher fluctuation on the left lateral region (black marker) in the oxidized (dark yellow) state, compared to the control (bright yellow) state.

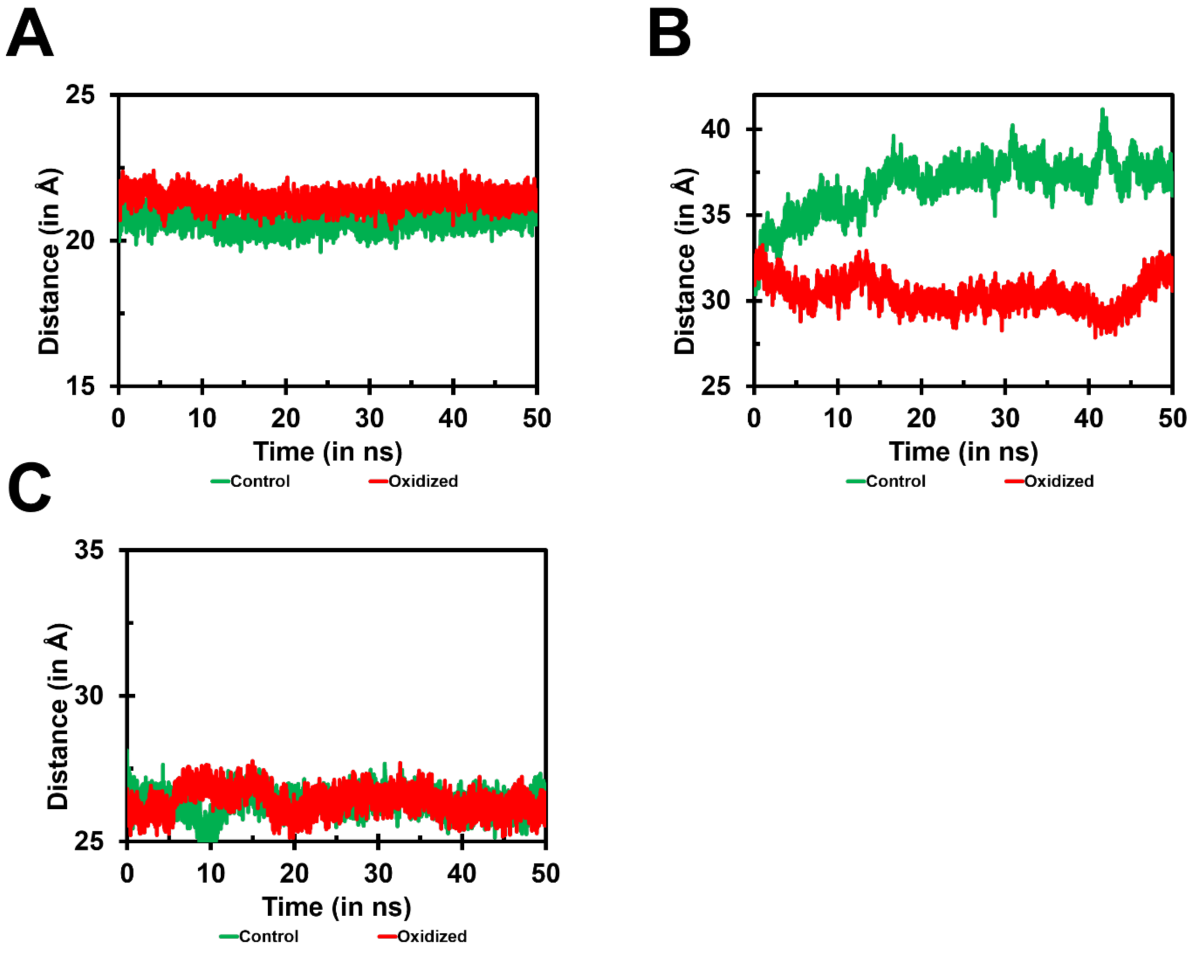

Figure S8 - Distance analysis between Electron transfer groups in the apo structure of CIII. **A** – Distance analysis between the two heme groups, b_L and b_H, housed in the MT-CYB subunit of the apo structure. **B** – Distance analysis between the 2[Fe-S] of UQCRFS1 and heme group from CYC1 of the apo structure. **C** - Distance analysis between the b_H of MT-CYB subunit and 2[Fe-S] of UQCRFS1 of the apo structure. Control state and oxidized state are indicated by green and red, respectively, in B-D.
